# Improvements in Dentistry

**Published:** 1893-11

**Authors:** F. J. S. Gorgas


THE
AMERICAN JOURNAL
—OF—
DENTAL SCIENCE.

VOL. XXVII. BALTIMORE, NOVEMBER, 1893. NO. 7.

ARTICLE I.

### IMPROVEMENTS IN DENTISTRY.

BY F. J. S. GORGAS, M. D., D. D. S.

The Greek historian, Herodotus, informs us that the Egyptians practiced the dental art; and in his second book he states that the art and practice of medicine was divided among the Egyptian priesthood, each physician and surgeon applying himself to one class of disease only; some to the head, others to the eye and others to the teeth; and, judging from the dental work found in some of the tombs of Egypt, we may conclude that the practitioners of that time were comparatively learned and highly proficient in one branch at least of dental art.

Among the Hebrews Moses legislated his famous law of a 'tooth for a tooth,' and the one who broke his fellow-man's tooth had to pay the sufferer a sufficient amount of money to have the substitute inserted. The Talmudical folk lore says: "If a man dreams that his false teeth have fallen out it is an omen that his children will soon die."

The Talmud allowed Jewish women "to go out on the Sabbath with their false golden or silver teeth." The Chinese of mediæval times appeared to be skilled enough to preserve the teeth. A Chinese manuscript deposited in the French Academy of Science gives much information concerning dental practice in ancient China and asserts that their dentists possessed a wonderful powder for the painless extraction of teeth. Even the Arabs paid great attention to their teeth. An Arabian general under Mohammed was slain, and his body could only be identified by means of his false teeth. The science of dental surgery was carried from Egypt to Greece. Homer tells us that Esculapius, who lived 1250 B. C., used a narcotic to produce insensibility when performing such an operation as tooth-drawing, and the latter was the first Grecian who filled the teeth.

Cicero gives to the third son of Æsculapius the credit of inventing an instrument for the extraction of diseased teeth. The Temple of Delphi contains a pair of leaden forceps for the extraction of teeth, which date back 2,000 B. C., and the 10th of the celebrated Greek Laws of the 12 tables allowed that any gold used to fasten the teeth might be burned with the body. The Greeks wore false teeth made of sycamore wood, which were fastened to the adjoining natural ones by ligatures of gold or silver, and decayed teeth were filled with a clay-like substance which became very hard and durable. Among the early Romans, Hippocrates treated of the teeth in his investigations of all branches of medicine, and gave many directions concerning the manner in which the dentition of the first set of teeth should be managed. The famous Martial, who lived in the first century B. C., speaks of a Roman dentist, Calcellius, as "in the habit of fastening as well as extracting teeth."

The instruments for dental and surgical purposes which are to be seen in the museums of Europe, together with the beautiful specimens of Etrurian and Phœnician dentistry, are very striking illustrations of the ability of the early dentists. Filled teeth, crown and bridge-work have been exhumed in various parts of Italy, Greece and

Egypt, and are now on exhibition at the Columbian Worlds Fair. Among the Etrurians dental science was studied and practiced as a branch of medicine, but, being imitative rather than creative, their art bore the marks of the Egyptian and the Grecian. Much has been claimed for the success of modern crown and bridge work, but the prehistoric dentists did the same ingenious work centuries ago. What the modern dentist can claim, however, is improvement in its construction and application. The Roman dentists filled teeth with lead, amalgam and gold and a fusible metal, as the tombs of the northern part of Italy proves, but it was only the nobles who were fortunate enough to receive the advantages of dental operations. During the Dark Ages all sciences and arts were completely neglected for the period of 1,000 years, when the surgical portion of dentistry and medicine was delegated to the barber and blacksmith and the prosthetic branch of dentistry to the skilled jewelers of those days, and it was not until late in 1700 that dental science came again into the hands of men who began a thorough study of its principles, and for many years after this period its scientific practice was limited to a few intelligent men, to whom the credit is due of rescuing it from the downtrodden trades and elevating it to its former and present dignity.

The crude extracting instruments of early days were at this period greatly improved, but still in accordance with the mistaken idea that force alone was necessary, and not such movements as were required by the anatomy of the parts. The key of Gazengeot succeeded the crude levers, and it was not until the latter part of the 18th century that the present style of forceps were invented. From 1835 to the present time many improved instruments for the removal of teeth from the jaws have been adopted, and to the late Drs. Chapin A. Harris and Edward Maynard, the former of this city, is due the credit, of originating many of the improved forms of forceps. Since their day, however, while the general forms remain, the shape of the edges of the beaks of these instruments, as well as of the beaks themselves, have undergone considerable change, thereby rendering their adaptation to the teeth more perfect and the use of the gum lancet less frequent, thus preventing much of the pain of such operations.

Although the use of general anæsthetics was known to the ancient Dioscorides, a Greek physician having administered mandrake—stropa mandragora—and morion, the former agent also being referred to by Pliny and Apulcius, and the Chinese and Hindoos using Indian hemp—cannable indica—yet later the use of such agents appear to have been forgotten, or, if remembered, regarded as the wild fantasy of a barbarous age. It was not until within the present century that sulphuric ether, nitrous oxide gas, chloroform, bichloride of methyl, annyl and a number of other general anæsthetics were discovered. To practitioners of dentistry the discovery and application of such properties in ether and nitrous oxide is due. The fatality attending the use of general anæsthetics led to the application of local anæsthetics in dental practice, and at the present time comprise such agents as absolute ether, rhigolence, cocaine, and combinations of the latter with carbolic acid, pyreth rum, aconite, sulphuric acid, &c. The first of these local obtunders were ice and salt, spray of ether and rhigolene, in what was called the "freezing process," which, however, have given place to cocaine and applications which are applied by injections with the hypodermic syringe.

The use of lead followed the hard clay-like substance for filling teeth, which in turn gave way to pure tin, amalgam of silver tin and mercury and gold, the tin and gold being in the form of thin leaves of foil. The use of the hand-mallet for condensing tin and gold in filling teeth was practiced some 50 or more years ago, and this form of mallet is still in use, an assistant, often a female, assisting. The improved mallets, however, are the automatic and electric, which enable operators of the present day to restore teeth with gold which were less than a quarter of a century ago considered irreparable. Improvements have also been made in the nature of the amalgams employed for filling teeth, much purer metals being used and greater care in their combination being exercised. By the use of such amalgams teeth that, owing to their frailty, are unable to withstand the force required to condense gold properly are now rendered useful organs.

Great improvements have also been made in prosthetic dentistry. Artificial teeth were rudely inserted in early days by bands attaching them to adjoining natural ones. Later both teeth and base were carved out of ivory or other bone, and, as a consequence, but ill adapted for fit, comfort and use. Natural teeth, human and also those of animals,. were attached to bone bases. Still later metallic plates holding mineral teeth were used, either clapsed by bands to natural teeth remaining in the mouth, or in the case of full or entire dentures, held together on the jaws by means of spiral springs. At the present time artificial teeth, especially upper sets, are so well adapted to the mouth that the adhering force is atmospheric pressure, applied either by close adaptation, or by the aid of a vacuum cavity in the surface of the plate next to the plate. Lower sets when entire are so adapted by closeness of fit that attachment to the upper sets by means of the spiral springs is no longer necessary.

The form of work known as crown and bridge work although used by the Romans, has been so greatly improved that now many artificial teeth can be inserted without the aid of a plate covering the greater part of the roof of the mouth, provided certain natural teeth, or even roots of teeth, remain so as to form points of vantage. Very frail natural crowns which cannot be preserved by filling can be protected and rendered useful for mastication by covering them with entire or open-faced crowns of gold so adapted to the teeth that what remains of them is imperishable.

